# Orthostatic Blood Pressure Changes and Associated Factors Among Adults With Diabetes in Cameroon: A Cross‐Sectional Study

**DOI:** 10.1155/ijhy/5603821

**Published:** 2026-10-26

**Authors:** Clovis Nkoke, Ryan Awa, Denis Teuwafeu, Leslie Tasha Mbapah, Simeon Pierre Choukem

**Affiliations:** ^1^ Department of Internal Medicine and Pediatrics Faculty of Health Sciences University of Buea Buea Cameroon ubuea.cm; ^2^ Department of Internal Medicine Buea Regional Hospital Buea Cameroon; ^3^ Department of Internal Medicine Icahn School of Medicine at Mount Sinai New York USA mountsinai.org; ^4^ Department of Clinical Sciences Faculty of Medicine and Pharmaceutical Sciences University of Dschang Dschang Cameroon univ-dschang.org

**Keywords:** Cameroon, cardiovascular autonomic dysfunction, diabetes mellitus, exaggerated orthostatic pressor response, orthostatic hypertension, orthostatic hypotension

## Abstract

**Background:**

Orthostatic blood pressure abnormalities are common in people with diabetes mellitus (DM) and are associated with adverse cardiovascular outcomes. Data on orthostatic hypotension (OH), exaggerated orthostatic pressor response (EOPR), and orthostatic hypertension (OHTN) in patients with diabetes from sub‐Saharan Africa are limited.

**Methods:**

We conducted a multicenter cross‐sectional study among 339 ambulatory adults with DM attending three referral hospitals in Cameroon between November 2023 and April 2024. OH, EOPR, and OHTN were defined according to the 2023 American Autonomic Society/Japanese Society of Hypertension consensus statement. Prespecified multivariable logistic regression models identified factors independently associated with OH and OHTN.

**Results:**

The mean age was 60.3 ± 11.3 years, and 66.1% of participants were female. The prevalence of OH was 13.3% (95% CI, 9.8–17.4), EOPR 8.9% (95% CI, 6.1–12.4), and OHTN 8.6% (95% CI, 5.8–12.1). A history of diabetes‐related complications (adjusted odds ratio [aOR] 2.47, 95% CI 1.05–5.81; *p* = 0.038) and ever smoking (aOR 2.85, 95% CI 1.09–7.44; *p* = 0.032) were independently associated with OH. Increasing age (aOR 1.08 per year, 95% CI 1.03–1.13; *p* = 0.002) and dyslipidemia (aOR 2.79, 95% CI 1.11–7.06; *p* = 0.030) were independently associated with OHTN. Sensitivity analyses using the previous diagnostic criteria increased the estimated prevalence of EOPR to 16.5% and OHTN to 15.0.

**Conclusions:**

Orthostatic blood pressure abnormalities were common among adults with DM in this hospital‐based cohort in Cameroon. OH and OHTN were associated with different clinical factors, suggesting potentially distinct underlying mechanisms. Routine orthostatic blood pressure assessment may provide additional information for cardiovascular risk stratification in adults with diabetes, particularly in resource‐limited settings.

## 1. Introduction

Diabetes mellitus (DM) is a major contributor to cardiovascular morbidity and mortality worldwide and represents a growing public health challenge, particularly in low‐ and middle‐income countries where the prevalence continues to rise. In addition to accelerating atherosclerotic cardiovascular disease, diabetes is associated with microvascular complications and cardiovascular autonomic neuropathy, both of which adversely affect blood pressure regulation and increase the risk of cardiovascular events and premature death [1, 2].

Maintenance of blood pressure during standing requires rapid activation of the arterial baroreflex, resulting in coordinated increases in sympathetic activity, peripheral vasoconstriction, and heart rate to preserve cerebral perfusion. In people with diabetes, chronic hyperglycemia, autonomic neuropathy, endothelial dysfunction, impaired baroreflex sensitivity, and increased arterial stiffness may disrupt these compensatory mechanisms, leading to abnormal postural blood pressure changes [2, 3]. Consequently, both excessive decreases and excessive increases in blood pressure after standing may occur, reflecting different manifestations of autonomic and vascular dysfunction.

Orthostatic hypotension (OH) is the most extensively studied postural blood pressure abnormality and has consistently been associated with falls, syncope, cognitive impairment, chronic kidney disease, cardiovascular disease, and increased all‐cause mortality [3]. Conversely, increasing evidence suggests that excessive orthostatic blood pressure elevation is also clinically important. Exaggerated orthostatic pressor response (EOPR) and orthostatic hypertension (OHTN) have been associated with increased arterial stiffness, sustained hypertension, target organ damage, stroke, and adverse cardiovascular outcomes, suggesting that both extremes of orthostatic blood pressure regulation may identify individuals at increased cardiovascular risk [4].

Historically, inconsistent definitions of OHTN have contributed to considerable heterogeneity across epidemiological studies. To address this limitation, the American Autonomic Society and the Japanese Society of Hypertension published an international consensus statement recommending that an orthostatic increase in systolic blood pressure (SBP) of ≥ 20 mmHg be defined as an EOPR, whereas OHTN should be diagnosed only when this increase is accompanied by a standing SBP of ≥ 140 mmHg [4].

The prevalence of postural blood pressure abnormalities among people with diabetes varies according to age, duration of diabetes, and comorbidity burden. In a study of community‐dwelling older adults, Kocyigit et al. reported prevalences of 17.3% for OH and 7.2% for OHTN in the overall cohort, compared with 17.7% and 9.6%, respectively, among older adults with diabetes [5]. However, evidence from sub‐Saharan Africa remains scarce, and most studies have focused exclusively on OH while overlooking EOPR and OHTN. Furthermore, few studies have examined determinants of these orthostatic blood pressure phenotypes among African adults with diabetes.

Cameroon is experiencing a growing burden of diabetes and cardiovascular disease, yet data describing postural blood pressure abnormalities in adults with diabetes are limited. Improved characterization of these abnormalities may facilitate earlier recognition of autonomic dysfunction, enhance cardiovascular risk stratification, and inform routine blood pressure assessment in diabetes clinics.

Therefore, this study aimed to determine the prevalence of OH, EOPR, and OHTN among ambulatory adults with diabetes attending three referral hospitals in Cameroon. We further sought to identify factors independently associated with orthostatic OH and OHTN.

## 2. Materials and Methods

### 2.1. Study Design and Setting

A hospital‐based cross‐sectional study was conducted from November 1, 2023, to April 1, 2024, involving ambulatory adult patients with DM attending the diabetic clinics at three referral hospitals, namely Buea Regional Hospital (BRH), Laquintinie Hospital Douala (LHD), and Douala General Hospital (DGH) in Cameroon.

The BRH, a secondary‐level hospital in Buea, Southwest Region, Cameroon, has a diabetic unit with a nursing station, a physician’s office, nurses, four general practitioners, a nutritionist, and a cardiologist. Operating weekly, it treats about 18 patients per session, starting with counseling and vital sign measurement, then a physician examination. About 1000 diabetic patients are registered.

The LHD is a secondary‐level hospital in Douala, Littoral region, Cameroon. Its outpatient diabetic unit includes a nursing station and two doctors’ offices. Staff consists of a nurse and three endocrinologists. Consultations are held Monday through Friday, with about 12 patients seen each day. Each patient has their vital signs checked by a trained nurse and receives personalized counseling based on their needs.

The DGH, a tertiary‐level hospital in Douala, Littoral region, has an outpatient diabetic unit with a nursing station, two consultation offices, and a waiting room. Staff includes one nurse and two endocrinologists. Consultations occur daily, Monday through Friday, averaging 14 patients per day. Patients’ vital signs are measured before their consultation, and nurses trained in counseling occasionally offer guidance during waits. The three hospitals were purposively selected because they had established specialist diabetes clinics with sufficient outpatient volumes for recruitment and included referral facilities from two regions of Cameroon and both secondary‐ and tertiary‐level care settings.

### 2.2. Study Population and Sampling

We included all individuals aged 21 years and older with a clinical diagnosis of DM who granted informed consent. The lower age threshold of 21 years was selected because this was the legal age of adulthood used for independent informed consent in Cameroon. We excluded participants with an acute febrile illness within 48 h before the orthostatic challenge and pregnant women. Participants were sampled through consecutive sampling of eligible individuals. The Cochran formula was used to calculate the minimum sample size.

(1) n=Z2P1−PE2,

 
*n* = required minimum sample size
 
*P* = 0.159, the estimated proportion of OH in patients on hemodialysis, in a study by Patrice et al. (2022), in Cameroon [6].
 
*Z* = 1.96, the z‐score at a 95% confidence level
 
*E* = 0.05, the margin of error

At a 95% confidence interval (CI), *Z* = 1.96, with a margin of error of 0.05.

A minimum sample size of 206 participants was obtained, with a 10% nonresponse rate; the minimum sample size was 227, but we recruited 339 patients with DM. A minimum of 100 participants were recruited from each facility to ensure representativeness.

## 3. Study Procedure

### 3.1. Data Collection and Procedure

Data collection was carried out daily at the diabetic clinics of the three previously mentioned hospitals, using a standardized data collection form that was pretested in another facility by the research team on 10 patients with DM. The form gathered information on sociodemographic characteristics such as age, gender, smoking history, alcohol consumption, duration of diabetes, and treatment history when available. Additionally, anthropometric measurements, including abdominal circumference, height, and weight, were obtained, and body mass index (BMI) was calculated. Blood pressure readings were performed using an automatic sphygmomanometer on the left upper arm (Omron HEM‐7342T‐Z, Omron Healthcare, Inc., Lake Forest, IL, USA). The protocol for assessing OH and OHTN included measurements taken in the supine (recumbent) and standing (erect) positions. Supine blood pressures were recorded twice and averaged after five minutes of rest, while standing blood pressures were measured at one, three, and five minutes following assuming an upright position. The history of dyslipidemia and diabetes‐related complications was based on participant reports of a previous diagnosis and was confirmed from available medical records when possible. These histories were recorded by the study data collectors; no study‐specific diagnostic testing was performed solely to establish new diagnoses.

Definition of orthostatic blood pressure phenotypes is as follows:

Primary definitions followed the 2023 international consensus statement by Jordan et al. [4].

• OH: a sustained reduction in SBP ≥ 20 mmHg and/or diastolic blood pressure ≥ 10 mmHg within 3 min of standing.
• EOPR: an increase in SBP ≥ 20 mmHg on standing.
• OHTN: an EOPR accompanied by an upright SBP ≥ 140 mmHg.

Hypertension was defined as resting blood pressure ≥ 140/90 mmHg or current use of antihypertensive medication. Blood pressure measurement followed the European Society of Hypertension recommendations [7].

### 3.2. Study Variables

Outcome variables: OH, EOPR, and OHTN.

Predictor variables: Age, sex, duration of diabetes, fasting blood glucose, BMI, waist circumference, smoking history, hypertension, dyslipidemia, history of diabetes‐related complications, cardiovascular comorbidities, antihypertensive medications (angiotensin‐converting enzyme [ACE] inhibitors, angiotensin receptor blockers, calcium channel blockers, beta‐blockers, and diuretics), and antidiabetic therapies (metformin, sulfonylureas, insulin, and dipeptidyl peptidase‐4 [DPP‐4] inhibitors). The diabetes‐related complication variable was a composite indicating a history of one or more available microvascular or macrovascular complications; participants with any recorded complication were classified as having a complication. Individual complications were not modeled separately because their frequencies were too low for stable multivariable estimates, and no participant reported diabetic nephropathy. The composite was not entered simultaneously with its individual components in multivariable models.

### 3.3. Data Management and Analysis

Data were entered into Microsoft Excel 2016, cleaned, and analyzed using StataNow/SE version 18.5 (StataCorp LLC, College Station, TX, USA).

Categorical variables are presented as frequencies and percentages. Continuous variables are summarized as means ± standard deviations (SD) when normally distributed and as medians with interquartile ranges (IQR) or ranges when non‐normally distributed. Pearson’s chi‐square test was used to compare categorical variables, Student’s independent *t*‐test for normally distributed continuous variables, and the Wilcoxon rank‐sum test for skewed continuous variables. The prevalence of OH, EOPR, and OHTN was estimated with corresponding 95% CIs using the Wilson binomial method.

Two prespecified multivariable logistic regression models were constructed separately for OH and OHTN to identify factors independently associated with each outcome. Covariates were selected a priori based on clinical relevance and existing literature. Adjusted odds ratios (aORs) with 95% CIs were reported.

Model performance was evaluated using the Hosmer–Lemeshow goodness‐of‐fit test to assess calibration; receiver operating characteristic curve analysis with calculation of the area under the curve (AUC) to assess discrimination; link tests to evaluate model specification; and variance inflation factors (VIFs) to assess multicollinearity among predictor variables.

Sensitivity analyses were performed using the previous OHTN definition, incorporating either an SBP increase of ≥ 20 mmHg or a diastolic blood pressure increase of ≥ 10 mmHg on standing, with corresponding OHTN defined by these criteria in conjunction with a standing SBP of ≥ 140 mmHg. Agreement between the primary consensus definitions and the sensitivity definitions was also evaluated.

All statistical tests were two‐sided, and a *p*‐value < 0.05 was considered statistically significant.

### 3.4. Ethical Considerations

Ethical approval (Ref: 2024/2191‐11/UB/IRB/FHS) was obtained from the IRB of the Faculty of Health Sciences, University of Buea, and administrative authorization from the relevant institutions. Participants’ informed consent was obtained before recruitment, respecting their right to withdraw at any time without consequences to their care. Their identities and related patient information were kept confidential.

## 4. Results

### 4.1. Sociodemographic and Clinical Characteristics of Study Participants

A total of 339 adults with DM were included in the analysis. The mean age was 60.3 ± 11.3 years; 66.1% were female, and 57.8% were ≥ 60 years. Hypertension was present in 49.0% of participants, dyslipidemia in 17.7%, and 10.9% had a history of at least one recorded diabetes‐related microvascular or macrovascular complication. The median duration of diabetes was 5 [2–9] years; the mean fasting blood glucose was 171.7 ± 82.7 mg/dL; the mean BMI was 28.6 ± 4.8 kg/m^2^; and the mean waist circumference was 95.1 ± 8.2 cm. Mean baseline seated systolic and diastolic blood pressures were 141.4 ± 17.1 and 81.2 ± 8.1 mmHg, respectively, while corresponding standing blood pressures were 142.4 ± 16.3 and 83.0 ± 9.0 mmHg (Table 1).

**TABLE 1:** Sociodemographic and clinical characteristics of patients with diabetes in the study.

Variable	Categories	Overall (*N* = 339)	OH (*n* = 45)	OHTN (*n* = 29)
Sociodemographic characteristics		*n* (%)	*n* (%)	*n* (%)

Age (years)	Mean ± SD	60.34 ± 11.28	64.16 ± 8.69	67.35 ± 10.56
	< 40	15 (4.4)	00 (0.0)	00 (0.0)
	40–59	128 (37.8)	10 (22.2)	7 (24.1)
	≥ 60	196 (57.8)	35 (77.8)	22 (75.9)

Sex	Male	115 (33.9)	15 (33.3)	13 (44.8)
	Female	224 (66.1)	30 (66.7)	16 (55.2)

Smoking	Never	305 (90.0)	36 (80.0)	26 (89.7)
	Ever	34 (10.0)	09 (20.0)	03 (10.3)

Clinical characteristics				

Diabetes duration (years)	Median (IQR)	5 (2–9)	6 (2–11)	6 (2–11)

Fasting blood glucose (mg/dL)	Mean ± SD	171.74 ± 82.74	180.02 ± 106.60	142.52 ± 51.55

Waist circumference (cm)	Mean ± SD	95.13 ± 8.19	96.44 ± 9.65	95.28 ± 9.20

Body mass index (kg/m^2^)	Mean ± SD	28.61 ± 4.76	28.96 ± 4.68	28.45 ± 6.09
	Normal	57 (16.8)	06 (13.3)	07 (24.1)
	Overweight	153 (45.1)	21 (46.7)	09 (31.0)
	Obese	129 (38.1)	18 (40.0)	13 (44.8)

Hypertension	No	173 (51.0)	12 (26.7)	11 (37.9)
	Yes	166 (49.0)	33 (73.3)	18 (62.1)

Dyslipidemia	No	279 (82.3)	32 (71.1)	19 (65.5)
	Yes	60 (17.7)	13 (28.9)	10 (34.5)

Diabetes‐related complications	No	302 (89.1)	33 (73.3)	25 (86.2)
	Yes	37 (10.9)	12 (26.7)	04 (13.8)

ACE inhibitor	No	261 (77.0)	28 (62.2)	21 (72.4)
	Yes	78 (23.0)	17 (37.8)	08 (27.6)

ARB	No	320 (94.4)	42 (93.3)	25 (86.2)
	Yes	19 (5.6)	03 (6.7)	04 (13.8)

Diuretic	No	258 (76.1)	29 (64.4)	21 (72.4)
	Yes	81 (23.9)	16 (35.6)	08 (27.6)

Calcium channel blocker	No	206 (60.8)	21 (46.7)	14 (48.3)
	Yes	133 (39.2)	24 (53.3)	15 (51.7)

Beta blocker	No	331 (97.6)	43 (95.6)	28 (96.6)
	Yes	08 (2.4)	02 (4.4)	01 (3.4)

Insulin	No	254 (74.9)	32 (71.1)	22 (75.9)
	Yes	85 (25.1)	13 (28.9)	07 (24.1)

DPP‐4 inhibitor	No	285 (84.1)	36 (80.0)	24 (82.8)
	Yes	54 (15.9)	09 (20.0)	05 (17.2)

Biguanide	No	62 (18.3)	07 (15.6)	03 (10.3)
	Yes	277 (81.7)	38 (84.4)	26 (89.7)

Sulfonylurea	No	180 (53.1)	27 (60.0)	15 (51.7)
	Yes	159 (46.9)	18 (40.0)	14 (48.3)

*Note: n*, frequency, %; percentage; DPP‐4, dipeptidyl peptidase‐4.

Abbreviations: ACEi, angiotensin‐converting enzyme inhibitor; ARB, angiotensin receptor blocker; BP, blood pressure; DBP, diastolic blood pressure; OH, orthostatic hypotension; OHTN, orthostatic hypertension; SBP, systolic blood pressure.

### 4.2. Prevalence of Orthostatic Blood Pressure Abnormalities

OH was identified in 13.3% (95% CI 9.8–17.4). EOPR was observed in 8.9% (95% CI 6.1–12.4), while OHTN was present in 8.6% (95% CI 5.8–12.1). Overall, 21.8% (95% CI 17.5–26.6) of participants met criteria for either OH or OHTN, indicating a high prevalence of orthostatic blood pressure abnormalities.

### 4.3. Sociodemographic and Clinical Characteristics of Study Participants According to Orthostatic BP Changes

Compared with participants without OH, those with OH were significantly older (64.2 ± 8.7 vs. 59.8 ± 11.5 years, *p* = 0.015) and exhibited higher baseline systolic and diastolic blood pressures (*p* < 0.001). Participants with OH were also more likely to have a history of diabetes‐related complications (26.7% vs. 8.5%, *p* < 0.001), hypertension (73.3% vs. 45.2%, *p* < 0.001), dyslipidemia (28.9% vs. 16.0%, *p* = 0.035), and a history of smoking (20.0% vs. 8.5%, *p* = 0.017). Use of ACE inhibitors, calcium‐channel blockers, and diuretics was also more frequent among participants with OH.

Compared with participants without EOPR, those with EOPR were significantly older (66.9 ± 10.6 vs. 59.7 ± 11.2 years, *p* < 0.001) and had lower fasting blood glucose concentrations (141.2 ± 51.2 vs. 174.7 ± 84.6 mg/dL, *p* = 0.034). Baseline SBP was significantly lower (132.0 ± 10.1 vs. 142.3 ± 17.3 mmHg, *p* = 0.002), whereas standing SBP was markedly higher (157.7 ± 10.5 vs. 140.9 ± 16.0 mmHg, *p* < 0.001). Consequently, participants with EOPR demonstrated substantially greater orthostatic increases in systolic (+25.6 ± 6.0 vs. −1.4 ± 12.9 mmHg, *p* < 0.001) and diastolic blood pressure (+5.8 ± 5.8 vs. +1.4 ± 7.1 mmHg, *p* < 0.001). Dyslipidemia was more prevalent among participants with EOPR (33.3% vs. 16.2%, *p* = 0.019). No significant differences were observed with respect to sex, duration of diabetes, BMI, waist circumference, hypertension, smoking status, diabetes‐related complications, or antihypertensive and glucose‐lowering medication use.

Participants with OHTN were significantly older than those without OHTN (67.3 ± 10.6 vs. 59.7 ± 11.1 years, *p* < 0.001). They had lower fasting blood glucose concentrations (142.5 ± 51.6 vs. 174.5 ± 84.6 mg/dL, *p* = 0.047) and lower baseline SBP (*p* = 0.004) but higher standing SBP (*p* < 0.001). Dyslipidemia was more common among participants with OHTN (34.5% vs. 16.1%, *p* = 0.013), and angiotensin receptor blocker use was more frequent (13.8% vs. 4.8%, *p* = 0.045).

### 4.4. Factors Independently Associated With OH in the Study Participants

A history of diabetes‐related complications (aOR 2.47, 95% CI 1.05–5.81; *p* = 0.038) and a history of smoking (aOR 2.85, 95% CI 1.09–7.44; *p* = 0.032) were independently associated with OH. Hypertension demonstrated a borderline association with OH (aOR 2.52, 95% CI 0.98–6.49; *p* = 0.056). Age (aOR 1.01, 95% CI 0.98–1.05; *p* = 0.424), female sex (aOR 1.01, 95% CI 0.47–2.17; *p* = 0.982), diabetes duration (aOR 1.03, 95% CI 0.97–1.09; *p* = 0.397), dyslipidemia (aOR 1.22, 95% CI 0.55–2.70; *p* = 0.626), fasting blood glucose (aOR 1.00, 95% CI 1.00–1.01; *p* = 0.382), waist circumference (aOR 1.01, 95% CI 0.97–1.05; *p* = 0.554), ACE inhibitor use (aOR 1.06, 95% CI 0.46–2.45; *p* = 0.885), and diuretic use (aOR 0.85, 95% CI 0.38–1.91; *p* = 0.687) were not independently associated with OH (Table 2).

**TABLE 2:** Multivariable logistic regression analysis for factors associated with orthostatic hypotension (*N* = 339).

Variables	Category	Multivariable logistic regression			*p*‐value
		%	aOR	(95% CI)	
Age (years)		60.34 ± 11.28	1.01	(0.98–1.05)	0.424

Duration of diabetes (years)		5 (1–40)	1.03	(0.97–1.09)	0.397

Diabetes‐related complication	Yes	10.9	2.47	(1.05–5.81)	0.038
	No	89.1	1	Reference	

Hypertension	Yes	49.0	2.52	(0.98–6.49)	0.056
	No	51.0	1	Reference	

Dyslipidemia	Yes	17.7	1.22	(0.55–2.70)	0.626
	No	82.3	1	Reference	

Smoking history	Ever smoker	10.0	2.85	(1.09–7.44)	0.032
	Never smoker	90.0	1	Reference	

Fasting blood glucose (mg/dL)		171.74 ± 82.74	1.00	(1.00–1.01)	0.382

Waist circumference (cm)		95.13 ± 8.19	1.01	(0.97–1.05)	0.554

ACE inhibitor	yes	23.0	1.06	(0.46–2.45)	0.885
	no	77.0	1	Reference	

Diuretics	yes	23.9	0.85	(0.38–1.91)	0.687
	No	76.1	1	Reference	

*Note:* %; percentage, ±; mean and standard deviation.

Abbreviations: aOR, adjusted odds ratio; ACE, angiotensin‐converting enzyme; CI, confidence interval.

The multivariable model demonstrated acceptable discrimination (AUC = 0.723), good calibration (Hosmer–Lemeshow *p* = 0.398), no evidence of model misspecification (linktest _hatsq *p* = 0.411), and low multicollinearity (mean VIF = 1.30).

### 4.5. Factors Independently Associated With OHTN in the Study Participants

Increasing age (aOR 1.08 per year, 95% CI 1.03–1.13; *p* = 0.002) and dyslipidemia (aOR 2.79, 95% CI 1.11–7.06; *p* = 0.030) were independently associated with OHTN after multivariable adjustment. Female sex (aOR 0.57, 95% CI 0.24–1.32; *p* = 0.191), hypertension (aOR 1.02, 95% CI 0.41–2.52; *p* = 0.961), diabetes duration (aOR 0.97, 95% CI 0.90–1.05; *p* = 0.490), fasting blood glucose (aOR 0.99, 95% CI 0.99–1.00; *p* = 0.151), waist circumference (aOR 0.99, 95% CI 0.94–1.04; *p* = 0.684), and smoking history (aOR 0.65, 95% CI 0.17–2.51; *p* = 0.527) were not independently associated with OHTN. Table 3.

**TABLE 3:** Multivariable logistic regression analysis for factors associated with orthostatic hypotension (*N* = 339).

Variables	Category	%	Multivariable logistic regression		*p*‐value
			aOR	(95% CI)	
Age (years)		60.34 ± 11.28	1.08	(1.03–1.13)	0.002

Sex	Female	66.1	0.57	(0.24–1.32)	0.191
	Male	33.9	1	Reference	

Duration of diabetes (years)		5 (1–40)	0.97	(0.90–1.05)	0.490

Hypertension	yes	49.0	1.02	(0.41–2.52)	0.961
	No	51.0	1	Reference	

Dyslipidemia	yes	17.7	2.79	(1.11–7.06)	0.030
	No	82.3	1	Reference	

Smoking history	Ever smoker	10.0	0.65	(0.17–2.51)	0.527
	Never smoker	90.0	1	Reference	

Fasting blood glucose (mg/dL)		171.74 ± 82.74	0.99	(0.99–1.00)	0.151

Waist circumference (cm)		95.13 ± 8.19	0.99	(0.94–1.04)	0.684

*Note:* %; percentage, ±; mean and standard deviation.

Abbreviations: aOR, adjusted odds ratio; CI, confidence interval.

The model demonstrated acceptable discrimination (AUC = 0.747), satisfactory calibration (Hosmer–Lemeshow *p* = 0.080), no evidence of model misspecification (linktest _hatsq *p* = 0.970), and minimal multicollinearity (mean VIF = 1.14).

### 4.6. Sensitivity Analyses

Using the previous SBP‐or‐DBP definition, the prevalence of EOPR increased from 8.9% to 16.5%, while the prevalence of OHTN increased from 8.6% to 15.0%. All participants meeting the Jordan consensus definition also met the previous definition, with the latter identifying an additional 26 individuals with EOPR and 22 individuals with OHTN.

Multivariable analysis using the sensitivity definition yielded findings consistent with the primary analysis. Increasing age (aOR 1.06, 95% CI 1.02–1.09; *p* = 0.002) and dyslipidemia (aOR 2.97, 95% CI 1.41–6.27; *p* = 0.004) remained independently associated with OHTN, supporting the robustness of the primary findings. The sensitivity model demonstrated acceptable discrimination (AUC = 0.726), good calibration (Hosmer–Lemeshow *p* = 0.569), no evidence of model misspecification, and low multicollinearity (mean VIF = 1.14).

## 5. Discussion

In this multicenter cross‐sectional study of 339 ambulatory adults with DM attending three referral hospitals in Cameroon, orthostatic blood pressure abnormalities were common. EOPR and OHTN were 13.3%, 8.9%, and 8.6%, respectively. A history of diabetes‐related complications and a history of smoking were independently associated with OH, whereas increasing age and dyslipidemia were independently associated with OHTN. These findings suggested that both orthostatic hypotensive and pressor responses were clinically relevant among adults with diabetes, although they may reflect different underlying mechanisms. The prevalence of OH observed in this study was lower than the pooled estimate of approximately 24% reported in a meta‐analysis of adults with diabetes by Zhou et al. [8] but remained within the wide range reported across diabetic populations. Similarly, Gaspar et al. [9] reported substantially higher OH prevalence in both type 1 and type 2 diabetes over 10 years of follow‐up, whereas Kocyigit et al. reported an OH prevalence of 17.7% among older adults with diabetes [5]. Within sub‐Saharan Africa, Migisha et al. reported comparable frequencies of orthostatic blood pressure abnormalities among ambulatory adults with diabetes in Uganda [10], while Enwonwu et al. observed a lower prevalence among elderly Nigerians with diabetes [11]. Differences between studies likely reflect variation in participant age, diabetes duration, cardiovascular risk profile, antihypertensive medication use, and orthostatic blood pressure measurement protocols. Unlike studies conducted exclusively among frail older adults or high‐risk cardiovascular cohorts, our participants were ambulatory clinic attendees with a relatively shorter duration of diabetes, which may partly explain the lower prevalence of OH.

The prevalence of OHTN in this cohort was remarkably similar to the 9.6% reported by Kocyigit et al. [5] among older adults with diabetes despite important differences in geography and study populations. Conversely, Mesquita et al. reported a considerably higher prevalence among elderly patients with type 2 diabetes [12], while earlier studies by Yoshinari et al. and Wijkman et al. also described higher frequencies using broader definitions that incorporated diastolic blood pressure increases or lower diagnostic thresholds [13, 14]. These observations highlight the difficulty of comparing historical studies because definitions of OHTN have varied substantially over time. The present study, therefore, provides one of the first estimates from sub‐Saharan Africa based on internationally accepted consensus criteria.

Increasing age emerged as an independent factor associated with OHTN. This finding is consistent with previous studies demonstrating that aging is associated with progressive arterial stiffening, impaired baroreflex sensitivity, reduced vascular compliance, and increased sympathetic activation. Diabetes may further amplify these processes through autonomic neuropathy, endothelial dysfunction, and microvascular injury [2, 4, 15]. The predominance of older participants among those with OHTN therefore supports the concept that OHTN represents a manifestation of accelerated vascular aging superimposed on diabetes‐related autonomic dysfunction.

Dyslipidemia was independently associated with OHTN. Dyslipidemia contributes to endothelial dysfunction, vascular remodeling, and increased arterial stiffness, all of which may impair normal cardiovascular adaptation during postural change. The clinical relevance of this finding is supported by the recent systematic review and meta‐analysis by Pasdar et al., which demonstrated that systolic OHTN is associated with increased risks of all‐cause mortality, cardiovascular mortality, and cerebrovascular events [16]. Although causality cannot be inferred from our cross‐sectional study, these findings suggest that OHTN may serve as a marker of adverse vascular health rather than merely an exaggerated physiological response to standing.

Interestingly, hypertension and fasting blood glucose were associated with OHTN in unadjusted analyses but were no longer statistically significant after multivariable adjustment. This likely reflects confounding by age and other cardiometabolic factors. Resting hypertension is strongly correlated with arterial stiffness, antihypertensive medication use, and advancing age, reducing its independent contribution once these variables are considered simultaneously. Likewise, fasting blood glucose represents short‐term glycemic status and may not adequately reflect cumulative metabolic exposure or autonomic injury. Measures such as glycated hemoglobin or glycemic variability may better capture the chronic metabolic burden associated with orthostatic blood pressure dysregulation [15, 17].

The determinants of OH differed substantially from those of OHTN, supporting the notion that these represented distinct clinical phenotypes. A history of diabetes‐related complications remained independently associated with OH after adjustment, consistent with prior work linking OH in diabetes to a greater burden of microvascular and macrovascular complications and with the recognized relationship between diabetic autonomic dysfunction and end‐organ injury [2, 9]. Notably, diabetes duration itself was not independently associated with OH, suggesting that accumulated end‐organ damage may be a better indicator of autonomic dysfunction than disease duration alone.

A history of smoking was also independently associated with OH, although the relatively small number of smokers warrants cautious interpretation. Smoking promotes endothelial dysfunction, oxidative stress, arterial stiffness, and autonomic imbalance, providing biological plausibility for this association [18, 19]. While confirmation in larger cohorts is needed, these findings further support smoking cessation as an important component of cardiovascular risk reduction in adults with diabetes.

The clinical implications of these findings are important. Orthostatic blood pressure assessment is inexpensive, rapid, and readily incorporated into routine diabetes care. OH has consistently been associated with increased risks of falls, cardiovascular disease, stroke, heart failure, and mortality [20], whereas growing evidence indicates that OHTN similarly predicts adverse cardiovascular and cerebrovascular outcomes [4, 16]. Routine assessment of orthostatic blood pressure may therefore improve cardiovascular risk stratification by identifying patients with occult autonomic or vascular dysfunction that may not be detected by seated blood pressure measurement alone, particularly in resource‐limited settings.

## 6. Strengths and Limitations

This study included participants from three referral hospitals, employed standardized orthostatic blood pressure measurements, and was among the first studies from sub‐Saharan Africa to apply the contemporary consensus definition distinguishing EOPR from OHTN. Nevertheless, the cross‐sectional design precluded causal inference, and the hospital‐based sample from referral diabetes clinics in two regions may limit generalizability to community populations, primary‐care settings, and other regions of Cameroon. Measures of cardiovascular autonomic function, ambulatory blood pressure monitoring, glycated hemoglobin, and arterial stiffness were unavailable, limiting mechanistic interpretation. Dyslipidemia and diabetes‐related complications were based on participant reports with medical‐record confirmation when available rather than uniform study‐specific diagnostic testing, introducing the possibility of misclassification or under‐ascertainment. Individual diabetes‐related complications were uncommon, limiting stable complication‐specific multivariable analyses. Finally, the relatively small numbers of participants with EOPR and OHTN limited statistical power for more detailed subgroup analyses.

## 7. Conclusion

Orthostatic blood pressure abnormalities were common among adults with diabetes in this hospital‐based cohort in Cameroon. A history of diabetes‐related complications and smoking was associated with OH, whereas increasing age and dyslipidemia were associated with OHTN. These findings suggested that routine orthostatic blood pressure assessment may provide additional information for cardiovascular risk assessment in adults with diabetes, particularly in resource‐limited settings where simple, low‐cost assessment tools are valuable. Long‐term studies are needed to assess the cardiovascular, renal, and mortality effects of OH, EOPR, and OHTN in African populations with diabetes. They should also evaluate whether early detection and targeted management of these blood pressure conditions improve clinical outcomes.

## Funding

No funding was received for this manuscript.

## Conflicts of Interest

The authors declare no conflicts of interest.

## Data Availability

The data that support the findings of this study are available from the corresponding author upon reasonable request.
